# Ethylene and Nitric Oxide Involvement in the Regulation of Fe and P Deficiency Responses in Dicotyledonous Plants

**DOI:** 10.3390/ijms22094904

**Published:** 2021-05-05

**Authors:** María José García, Carlos Lucena, Francisco Javier Romera

**Affiliations:** 1 Department of Botany, Ecology and Plant Physiology, Campus de Excelencia Internacional Agroalimentario, Universidad de Córdoba, 14071 Córdoba, Spain; 2 Department of Biochemistry and Molecular Biology, Campus de Excelencia Internacional Agroalimentario, Universidad de Córdoba, 14071 Córdoba, Spain; b42lulec@uco.es; 3 Department of Agronomy, (DAUCO-María de Maeztu Unit of Excellence) Campus de Excelencia Internacional Agroalimentario, Universidad de Córdoba, 14071 Córdoba, Spain; ag1roruf@uco.es

**Keywords:** iron, phosphorous, nutrient deficiencies, Strategy I, ethylene, nitric oxide, S-nitrosoglutathione, acid phosphatase, ferric reductase, EIN3

## Abstract

Iron (Fe) and phosphorus (P) are two essential elements for plant growth. Both elements are abundant in soils but with poor availability for plants, which favor their acquisition by developing morphological and physiological responses in their roots. Although the regulation of the genes related to these responses is not totally known, ethylene (ET) and nitric oxide (NO) have been involved in the activation of both Fe-related and P-related genes. The common involvement of ET and NO suggests that they must act in conjunction with other specific signals, more closely related to each deficiency. Among the specific signals involved in the regulation of Fe- or P-related genes have been proposed Fe-peptides (or Fe ion itself) and microRNAs, like miR399 (P), moving through the phloem. These Fe- or P-related phloem signals could interact with ET/NO and confer specificity to the responses to each deficiency, avoiding the induction of the specific responses when ET/NO increase due to other nutrient deficiencies or stresses. Besides the specificity conferred by these signals, ET itself could confer specificity to the responses to Fe- or P-deficiency by acting through different signaling pathways in each case. Given the above considerations, there are preliminary results suggesting that ET could regulate different nutrient responses by acting both in conjunction with other signals and through different signaling pathways. Because of the close relationship among these two elements, a better knowledge of the physiological and molecular basis of their interaction is necessary to improve their nutrition and to avoid the problems associated with their misuse. As examples of this interaction, it is known that Fe chlorosis can be induced, under certain circumstances, by a P over- fertilization. On the other hand, Fe oxides can have a role in the immobilization of P in soils. Qualitative and quantitative assessment of the dynamic of known Fe- and P-related genes expression, selected ad hoc and involved in each of these deficiencies, would allow us to get a profound knowledge of the processes that regulate the responses to both deficiencies. The better knowledge of the regulation by ET of the responses to these deficiencies is necessary to properly understand the interactions between Fe and P. This will allow the obtention of more efficient varieties in the absorption of P and Fe, and the use of more rational management techniques for P and Fe fertilization. This will contribute to minimize the environmental impacts caused by the use of P and Fe fertilizers (Fe chelates) in agriculture and to adjust the costs for farmers, due to the high prices and/or scarcity of Fe and P fertilizers. This review aims to summarize the latest advances in the knowledge about Fe and P deficiency responses, analyzing the similarities and differences among them and considering the interactions among their main regulators, including some hormones (ethylene) and signaling substances (NO and GSNO) as well as other P- and Fe-related signals.

## 1. Introduction

Since the mid-twentieth century, a great increment in crop productivity has occurred all over the world due to the use of more productive varieties, fertilizers, herbicides and pesticides. The abusive use of some chemical products has led to the appearance of environmental problems, such as water eutrophication [1] and biodiversity losses [2].

In the most developed regions, such as EEUU, Europe and more recently in China, where a high agriculture intensification has been taking place in the last decades, the high P content in agricultural soils is becoming an environmental problem [3]. High P levels in soils increase the water eutrophication risk due to P losses by erosion and runoff [4]. On the other hand, in low resource supply agricultural systems, the crop yield is limited by P deficiency that affects up to 30% of the arable area all over the world [5]. Moreover, the price of phosphate fertilizers has considerably increased in recent decades due to the fast exhaustion of the phosphate reserves [6].

Iron (Fe) deficiency is also a worldwide problem in crop production on calcareous soils [7,8] and particularly for European fruit tree orchards [9]. In Spain, the problem is very important due to the high occurrence of calcareous soils. For instance, in the Ebro river basin area, an important agricultural area in Northeastern Spain, approximately 45,000 ha of orchards are affected [10], particularly peach trees (23,000 ha), one of the fruit trees most susceptible to Fe chlorosis [11]. Another important crop in Spain is the olive tree (*Olea europaea* L.). About 70% of the olive trees are grown on calcareous soils, which cause a significant decrease in their yield [12]. To avoid the negative effects of Fe deficiency in plants development and, consequently, on crop productivity, synthetic chelates are widely used by growers nowadays. This treatment entails a high economic cost [9,13] and some environmental problems.

Fe and P are two essential elements for plant growth and development [7]. Fe is necessary for important physiological processes such as photosynthesis, respiration and nitrogen assimilation [7]. In addition, Fe acts as a cofactor of several enzymes involved in removing reactive oxygen species, thus preventing the cellular damage caused by them [7,14]. Fe is also an essential nutrient for animals and humans, who can obtain it from plants.

P participates in a huge number of biological processes, among them, those related to energy use by the plant. It is part of the ATP molecule and of other cell components. It is involved in nucleic acid synthesis, photosynthesis, respiration, glycolysis, enzyme activation and deactivation, carbohydrates metabolism, nitrogen fixation and is part of the biological membranes [7,15].

## 2. Fe Acquisition by Dicotyledonous Plants

Plants have developed different strategies to improve Fe acquisition from soil. Strategy II, used by grasses, is based on the release of Fe chelating agents, called phytosiderophores, to the medium [16]. Strategy I, used by all higher plants except grasses [16], is characterized by the necessity of reducing Fe^3+^, the most abundant form in soil, to Fe^2+^, prior to its absorption. This reduction is mediated by a ferric reductase located in the plasma membrane of the epidermal root cells and encoded by *FRO2* in *Arabidopsis* [17]. Once Fe^3+^ has been reduced, it is transported into the cells by a Fe^2+^ transporter encoded by *IRT1* in *Arabidopsis* [18].

This review will focus on Strategy I plants because they are more susceptible to suffer Fe deficiency than Strategy II plants. On the other hand, dicotyledonous (Strategy I) plants present many similarities between their Fe and P deficiency responses.

When dicotyledonous plants suffer from Fe deficiency, they induce physiological and morphological changes in their roots, known as “Fe deficiency responses”, aimed to improve its mobilization and uptake. The main physiological responses are: enhanced ferric reductase activity, enhanced Fe^2+^ transport, and rhizosphere acidification, due to the upregulation of FERRIC REDUCTATE OXIDASE (*FRO*), IRON-REGULATED TRANSPORTER (*IRT*) and H^+^-ATPase (*HA*) genes [19,20,21,22]. Other relevant physiological responses are the increased synthesis and/or release of organic acids, phenolic compounds, such as coumarins and flavins, which can act as chelating and reducing Fe agents, improving its solubility for plants [23,24,25,26,27]. In addition, coumarins could also increase Pi availability for plants, by releasing it from Fe oxides. Some genes related to coumarin synthesis notably increase their expression under Fe deficiency [23]. Among them are feruloyl CoA hydroxylase 1 (*F6′H1*), which is involved in esculetin synthesis [28], and scopoletin 8 hydroxylase (*S8′H*), involved in fraxetin synthesis [29,30]. Highly oxygenated coumarins are excreted by the ATP-binding cassette (ABC) transporter ABCG37 (also named PDR9). In the *abcg37* mutants, coumarins exudation was compromised [31]. Morphological responses are aimed to increase the contact surface of roots with soil and include development of subapical root hairs, of cluster roots (also named proteoid roots) and of transfer cells [32,33]. Physiological responses are generally located in the subapical regions of roots [32].

The master regulator of most Fe deficiency responses in *Arabidopsis* is the FER-LIKE Fe DEFICIENCY-INDUCED TRANSCRIPTION FACTOR (FIT), a basic helix-loop-helix (bHLH) transcription factor (TF) which is induced in Fe-deficient roots [34,35,36]. Both *FRO2* and *IRT1* are transcriptionally activated by FIT, which needs to interact with some Ib bHLH subgroup TFs, such as bHLH38, bHLH39, bHLH100 and bHLH101, to be effective. These Ib bHLH subgroup TFs also increase their expression under Fe deficiency conditions [34]. The Ib bHLH subgroup is subjected to a positive regulation at transcriptional level by other bHLH TFs belonging to the IVc subgroup (bHLH34/104/105/115), which interacts with the phosphorylated form of Upstream Regulator of IRT1 (URI) [37]. The phosphorylated form of URI accumulates under Fe-deficient conditions. Under Fe-sufficient conditions, the phosphorylated URI and the IVc bHLH subgroup are subjected to proteasomal degradation depending on the E3 ligase BRUTUS (BTS) [37,38,39]. BTS has also been suggested to interact with the bHLH TF POPEYE (PYE), which positively regulates several responses to Fe deficiency [38]. Besides bHLH TFs, FIT can also interact with other TFs, like ETHYLENE INSENSITIVE3 (EIN3) and ETHYLENE INSENSITIVE3-LIKE1 (EIL1), two TFs involved in the ethylene (ET) transduction pathway, demonstrating a direct molecular link between ET signaling and FIT [40,41].

Besides *FRO2* and *IRT1*, FIT also positively regulates other physiological responses, such as acidification and coumarin synthesis and excretion. In the case of acidification, it has been shown that *AHA7* and *AHA2* (encoding H^+^-ATP ases) expression depends on FIT [42,43,44]. In the case of coumarins, FIT has been involved in the upregulation of *S8′H*, *F6′H1*, *BGLU42* (β-glucosidase) and *PDR9*, all of them involved in coumarin synthesis and/or release [23,42]. FIT mainly regulates the accumulation of the catechol type coumarins sideritin glucoside and fraxin, as demonstrated by the absence of Fe deficiency-induced accumulation of *S8′H* transcripts in *fit* plants [42]. Although FIT regulates *F6′H1* expression, *fit* mutants still produce appreciable levels of scopolin, scopoletin, and esculin, indicating that other factors are involved in the control of coumarin synthesis upstream of fraxin [45]. The regulation of *BGLU42* and *PDR9* expression is mediated by FIT through the MYB72 TF [46].

In recent years, the dynamic of utilization of the Fe pool stored in cell walls and the endodermal suberization have been emerging as Fe deficiency responses. Cationic nutrients bound to negatively charged cell wall components, mainly pectin and hemicellulose, represent a sink for this kind of nutrients [47]. Under Fe deficiency conditions, the Fe pool in the cell walls is remobilized, leading to an increase of soluble Fe in roots [48]. Thereby, modification of the cell wall composition could contribute to improve Fe availability. Under Fe deficiency conditions, the methylation degree of pectin and hemicellulose is lower, increasing the negative charges of the cell wall and improving the Fe reservoir in the root apoplast [49]. Meanwhile, endodermis suberization can affect nutrient acquisition in a selective manner [46]. Very recently, it has been shown that this is a dynamic process depending on a plant´s nutritional status. Under Fe-deficient conditions, endodermis suberization is delayed and it is also diminished in the Fe-deficient mutant *irt1* [50].

## 3. P Acquisition by Dicotyledonous Plants

Plants acquire P from the soil as inorganic phosphate (Pi). The available Pi concentration in non-fertilized soils is frequently below the one necessary for optimal maintenance of crops [51,52]. This is due to several Pi properties, such as low mobility; precipitation in insoluble forms with cations such as magnesium, calcium and metals; fixation to organic compounds [53]; and Fe oxides surface adsorption [54].

Pi is absorbed from soil through phosphate transporters, encoded by PHOSPHATE TRANSPORTER 1 (*PHT1*)-like genes, which are induced by P deficiency and have a great potential to improve P acquisition [55]. In *Arabidopsis,* the main Pi transporters implicated in its acquisition from the medium are *PHT1;1* and *PHT1;4*, also named *PT1* and *PT2* [15,56,57,58,59]. Both transporters are located in subapical root hairs [60,61].

When plants are grown under P deficiency conditions, they induce several physiological and morphological responses, mainly in their roots, to improve acquisition. The main physiological responses are the increase of phosphate transporters, the acidification of the rhizosphere, and the exudation to the rhizosphere of phosphatases, encoded by genes like *PAP17* in *Arabidopsis* (also named *ACP5*, “Acid Phosphatase”), and organic acids [51,58,59,62]. In most soils, Pi is not accessible for plants, but it can be solubilized with protons and organic anions. Many works have shown that most species are able to exudate organic acids to the rhizosphere that help to solubilize insoluble P compounds [63,64]. On the other hand, organic P from soil is not available for plants, unless it is hydrolyzed and mineralized to Pi by the action of phosphatases, which facilitate organic P assimilation from the rhizosphere [65,66]. 

The regulation of phosphate transporter genes, like *PHT1;1* and *PHT1;4*, is mediated by several TFs, such as the PHOSPHATE RESPONSE (PHR1) and the PHOSPHATE TRANSPORTER TRAFFIC FACILITATOR1 (PHF1) [67]. PHR1 regulates the expression of about 60% of the P deficiency-related genes [68]. It is induced by P deficiency and is regulated by the EIN3/EIL1 TFs [69]. Several P deficiency-related genes not regulated by PHR1 can be regulated by PHL (PHR1-like) TFs [68]. P deficiency-related genes are negatively regulated by SPX proteins, for which three isoforms have been found, and by the bHLH32 TF [70]. Under P sufficient conditions, SPXs interact with PHR1, avoiding its binding to the promoter region of the P-regulated genes [71,72]. However, the negative regulation of the P deficiency responses mediated by the bHLH32 TF seems to be independent of P levels [70].

Recently, it has been reported that coumarin profiles are also altered in root exudates of P-deprived plants [73]. These authors observed higher accumulation of a subset of coumarin derivatives, whereas the abundance of others, mainly highly oxygenated coumarins, was decreased when compared to exudates of P replete roots. Conversely, in Fe-deficient growth conditions, accumulation of all coumarins is promoted, mainly the highly oxygenated coumarins or methylated derivatives [23,74,75]. It has been suggested that coumarins are involved in the Fe accumulation induced by Pi deficiency [73]. Fe accumulation then promotes structural changes in root architecture [76]. Dihydroxyscopoletin is one of the main coumarins secreted under Fe deficiency conditions while its levels decreased under Pi deficiency [23]. It has been suggested that this could be a strategy of plants to decrease Fe acquisition and restore the growth of the primary root. In a very recent work, it has been proposed a key role for the Mitogen-Activated Protein Kinase 6 (MPK6) to coordinate the activity of meristematic root cells in response to Fe and P availability to optimize the primary root growth [77].

The role of several key regulators of Fe and P deficiency responses in coumarin biosynthesis has been studied in a very recent work [45], showing a key role of Fe-related regulators (bHLH104, BTS, PYE) and P-related regulators (PHR1, PHL1 and SPX) in the regulation of coumarin accumulation under both Fe and P deficiency conditions. The results obtained led the authors to draft a tentative model integrating the possible interactions between Fe- and P-related regulators in coumarin synthesis [45].

Finally, another important response to P deficiency is the internal phosphate remobilization, which includes Pi vacuolar release and membrane phospholipids change by sulpholipids and galactolipids [63,64,78]. In this function, the *Arabidopsis* PHT1;5 transporter plays a critical role in mobilizing Pi from P source to sink organs. The WRKY75 TF has been involved in the regulation of *PHT1;5* expression since relative *PHT1;5* transcript levels in a WRKY75-RNAi line were reduced under P-sufficient and P-deficient conditions [79].

Morphological responses to P deficiency include primary root growth inhibition, development of lateral roots, of root hairs, of transfer cells [72,80,81,82] and an increase of the root/shoot ratio [83,84]. Some plant species develop cluster roots when grown under P (also under Fe) deficiency conditions [63,64,72,82]. These kinds of roots improve the absorption of these nutrients and can excrete 20-fold more organic acids than normal roots [85,86]. Lupin (*Lupinus albus*) is a model plant used to study cluster roots under P deficiency conditions [85,87].

## 4. Fe and P Nutrition Interactions. A Great Opportunity to Improve the Nutrition of Both Elements

Several works have shown that excess P fertilization negatively affects Fe acquisition by plants and vice versa. In 1992, Romera et al. [88] found a decrease of the ferric reductase activity in sunflower and cucumber roots when the P concentration in the nutrient solution was increased in the presence of bicarbonate. On the other hand, Misson et al. [89] and Hirsch et al. [53] showed that Fe is one of the most accumulated metals in chloroplasts of plants grown under P deficiency. In parallel to this accumulation, the expression of several genes related to Fe homeostasis and storage in *Arabidopsis*, such as *NAS3* (Nicotianamine Synthase 3) or *FER1* (Ferritin 1), were induced. Bournier et al. [90] found that the PHR1 TF binds the *FER1* gene promoter (encoding a ferritin), showing a direct connection between P and Fe homeostasis [52].

Ward et al. [91] showed, in *Arabidopsis* plants grown under P deficiency conditions, an important inhibition of the primary root growth because of a high Fe accumulation, which becomes toxic. Fe accumulation promotes changes, induced by P deficiency, on the root system architecture [76]. In fact, the decrease of dihydroxyscopoletins during P deficiency, which are secreted mainly in response to Fe deficiency [23], could be a strategy to reduce Fe absorption and restore, in this way, the primary root growth [73].

Recently, Sánchez-Rodríguez et al. [92,93] have shown that phosphate fertilization alters Fe availability in soils and can aggravate Fe deficiency-induced chlorosis in susceptible plants. On the other hand, Fe oxides play an important role in controlling the P availability in soils, since Pi is adsorbed on their surface. Coumarins, which are excreted under Fe deficiency [73], could increase Pi availability to plants, by releasing it from the Fe oxides. Very recently, Chutia et al. [45] showed that Fe and Pi availability can modify the regulation of Fe/Pi deficiency-induced coumarin profiles through Fe/Pi deficiency response regulators (see previous Section).

Fe/P interactions are, therefore, numerous and varied and can determine the efficiency of Fe and P nutrition in plants. The control of the Fe/P interactions could be a new and effective way to improve the P and Fe nutrition of crops [91]. However, it is necessary to deeply know the mechanisms underpinning their interactions to ensure the success and to obtain more efficient genotypes in the acquisition of both nutrients.

## 5. Role of ET and NO in the Regulation of Fe and P Deficiency Responses. Similarities and Differences

Fe and P deficiency responses, which enable plants to improve their ability to acquire these nutrients, show many similarities [58,59,94]. Several responses to both deficiencies are similar, like rhizosphere acidification, enhanced synthesis of organic acids and coumarins, and development of subapical root hairs and proteoid roots. Furthermore, both kinds of responses share common regulators, such as ET and nitric oxide (NO), which increase their production in Fe-deficient roots as well as in P-deficient roots [33,81,82,95,96,97,98,99,100,101].

### 5.1. ET and NO Involvement in the Regulation of Physiological and Morphological Responses to Fe Deficiency

Results obtained by Romera’s group and Lamattina’s group showed for the first time the implication of ET/NO in the activation of physiological responses to Fe deficiency and in most of the Fe-related genes associated with them [22,102,103,104,105,106,107]. ET/NO have also been involved, along with auxin and other signaling substances, in the development of most of the Fe deficiency morphological responses [reviewed in 33 and 81]. Romera’s group was also the first one to study the ET/NO interactions in such a regulation [107,108,109]. Furthermore, this group, along with Dr. Yeh’s group, has also paved the way to study the relationship between NO and *S*-nitrosoglutathione (GSNO) in the regulation of Fe deficiency responses please check the following Section [110,111,112]. NO can react with glutathione (GSH) to produce GSNO, the main NO reservoir in plants [113]. GSNO levels are regulated by the GSNOR enzyme [112]. The *GSNOR1* gene, encoding this enzyme in *Arabidopsis*, is upregulated under Fe deficiency [112]. GSNO and NO have been found to play key roles in the regulation of Fe deficiency responses and other abiotic and biotic stress responses [34,106,107,108,110,111,112,114,115,116].

ET and NO have also been involved in the regulation of the cell wall composition and in the dynamic of the endodermal cells suberization, respectively. Fe deficiency increases the synthesis of putrescine, a natural polyamine that triggers a NO burst, which acts as a positive regulator of the solubilization of cell wall-bound Fe. The beneficial effect of putrescine on cell wall Fe utilization is lost in NO synthesis mutants: NO-associated 1 mutant (*noa1*) and nitrate reductase mutants 1 and 2 (*nia1nia2*) [117]. This beneficial effect is also lost in the hemicellulose production mutant Xyloglucan endotransglucosylase/hydrolase 31 mutant (*xth31*) [117] and in the reduced putrescine (Put) content mutant *adc2* [46]. ET acts as a negative regulator of suberization through the inhibition of the suberin production and by promoting its degradation [46].

### 5.2. ET and NO Involvement in the Regulation of Physiological and Morphological Responses to P Deficiency

For decades, the ET involvement in the regulation of P deficiency responses has been limited to its role in the inhibition of the primary root growth and in the development of lateral roots and root hairs [96,118,119,120]. Nowadays, there are experimental results showing that ET also plays an important role in the regulation of physiological responses to P deficiency [57,99,121,122,123]. Lei et al. [121] showed that the ET insensitive *Arabidopsis* mutants *etr1* and *ein2-5* present a lower expression of the genes encoding the P transporters *PT1*(*PHT1;1*) and *PT2*(*PHT1;4*). On the contrary, *Arabidopsis hps2* (*ctr1* homolog), a constitutive ET signaling mutant [124], displays enhanced responses to P deficiency [121]. ET has also been involved in *PHT1;5* regulation [57,79]. Finally, very recently it has been shown that *PHR1* expression is induced by ACC (ET precursor) [69].

In other plant species, such as *Medicago falcata*, results also support a role for ET in the regulation of P deficiency responses [125]. The expression of *MfPT1* and *MfPT5*, encoding P transporters, and of *MfPAP1*, encoding an acid phosphatase, was clearly inhibited by the ET synthesis inhibitors aminoethoxyvinyl glycine (AVG) and cobalt (Co^2+^). On the other hand, the treatment with the ET precursor ACC produced an increase of the expression of those genes in plants grown under P-sufficient conditions [125].

To date, the role of NO, GSNO and GSNOR in the regulation of physiological responses to P deficiency has been poorly studied [101]. However, in a recent work [126] it has been shown that NO induces the expression of the P transporter *OsPT2*. On the other hand, and as occurs under Fe deficiency, the *GSNOR1* gene is also upregulated under P deficiency [112].

Since many years, ET has been involved in the regulation of morphological responses to Fe and P deficiency, like subapical roots hairs, proteoid roots and transfer cells reviewed in [33,81,104]. For example, Zaid et al. [95] found that proteoid root formation in *Casuarina glauca*, induced by Fe deficiency, was inhibited by treatments with several ET synthesis and action inhibitors, like aminooxyacetic acid (AOA), cobalt and silver thiosulfate (STS), while the ET precursor ACC induced the development of proteoid roots in Fe-sufficient plants. These results clearly show a role for ET in this typical morphological response to either Fe or P deficiency.

In addition to ET, NO has been involved in the regulation of morphological responses to Fe and P deficiency [100,101,106]. The development of proteoid roots in lupine plants growing under Fe and P sufficiency, and the expression of *LaSCR1 and LaSCR2*, two essential genes related to the development of proteoid roots, were induced by GSNO (NO donor) treatment [97,127]. On the other hand, the development of proteoid roots, and the expression of the genes previously mentioned, were inhibited in Fe- or P-deficient plants by treatment with cPTIO (2-4-carboxyphenyl-4, 4, 5, 5-tetramethylimidazoline-1-oxyl-3-oxide), a NO chelating agent [127].

## 6. Mechanisms for the Induction of Fe- and P-Related Genes by ET and NO

**Figure 1:**
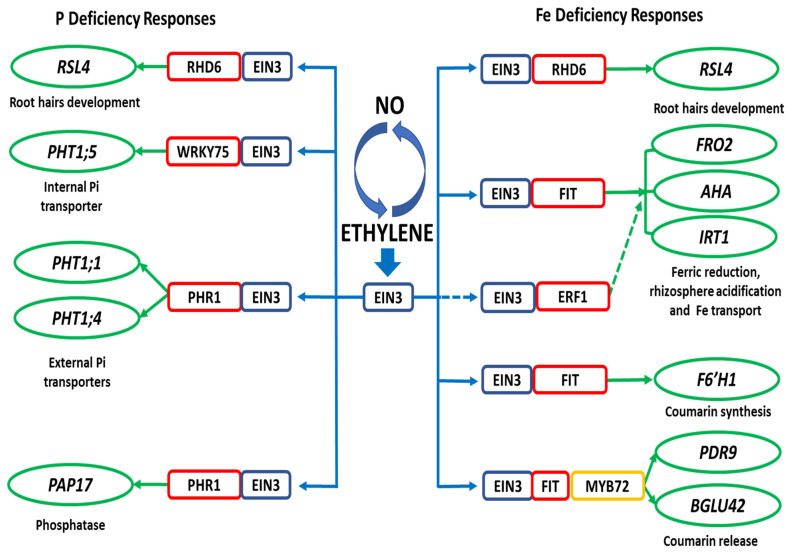
Working model proposed to explain the regulation of P and Fe deficiency responses by ET through EIN3. EIN3 (also EIL1) interacts with the PHR1 and WRKY75 TFs to regulate some P deficiency responses, such as expression of the internal (*PHT1;5*) and external (*PHT1;1* and *PHT1;4*) Pi transporters, and *PAP17* (acid phosphatase). EIN3 (also EIL1) interacts with the FIT TF to regulate some Fe deficiency responses, such as expression of *FRO2* (ferric reductase), *IRT1* (Fe transporter), *F6´H1*, *BGLU42* and *PDR9* (genes related to coumarin synthesis and release; these latter ones activated through the MYB72 TF). EIN3 can also interact with the ERF1 TF to regulate *FRO2* and *IRT1* expression. Finally, EIN3 can also interact with RHD6 to modify the expression of RSL4 and promotes root hair elongation either under P deficiency or Fe deficiency. Based on [33,40,41,46,69,96,99,131,135,136,137].

The mechanisms by which ET and NO regulate the responses to both deficiencies are not completely known, although some advances have been achieved in the last years. For ET, it has been found that EIN3/EIL1 are common key TFs involved in the regulation of physiological and morphological responses to Fe and P deficiency (Figure 1). In 2011, Lingam et al. [40] identified EIN3 and EIL1 in a screen for direct FIT interacting partners and validated their physical interaction in planta. Based on their results, they concluded that EIN3/EIL1 are required for full-level FIT accumulation [40]. EIN3/EIL1 physical interaction with FIT avoids its proteasomal degradation. In this way, ET, through EIN3/EIL1-FIT interaction, regulates the Fe acquisition genes *FRO2* and *IRT1*. *FIT* is also regulated at the transcriptional level through EIN3/EIL1 [41]. *FIT* expression, and consequently *IRT1* and *FRO2* expression, is diminished in the *yid1* and *med25* mutants under Fe-deficient conditions. YID1 encodes the *Arabidopsis* Mediator complex subunit MED16, which interacts with MED25 and subsequently with EIN3/EIL1 to regulate *FIT* transcription [41]. Very recently, García et al. [98] found that several Fe and P deficiency responses can also be induced in the *Arabidopsis* ethylene insensitive *ein3eil1* mutant, which suggests the existence of additional EIN3/EIL1-independent alternate routes for the activation of such responses. However, some of these Fe and P deficiency responses are impaired in *Arabidopsis* ethylene insensitive *ein2* mutants, which suggests the participation of the EIN2 protein, a critical component of the canonical ethylene signaling pathway, in such a regulation [128].

*FRO2* and *IRT1* expression may also be regulated in a FIT independent manner through the ERF1 TF, a downstream component of the ET signaling pathway directly regulated by EIN3 [129,130,131]. Other Ethylene Response Factors (ERFs) involved in the regulation of Fe deficiency responses are ERF4 and ERF72, which have been described as negative regulators of the responses [132,133,134]. *Arabidopsis thaliana erf4* and *erf72* mutants present higher chlorophyll content and lower expression of the chlorophyll degradation gene *CLH1* than wild-type plants. Ferric reductase activity and *IRT1* and *HA2* expression in roots are also significantly higher in these *Arabidopsis* mutants, or in *MxERF4* silencing lines, than in wild-type plants [132,133,134]. On the contrary, transient overexpression of *ERF4* results in rapid chlorophyll degradation in the leaves of *Nicotiana tabacum* and *CLH1* upregulation. Based on yeast one-hybrid experimental results, authors concluded that *ERF4* binds directly to the *CLH1* and *ITR1* promoters [133].

Coumarin synthesis is also regulated by ET through EIN3/EIL1, since *F6´H, BGLU42* (both involved in coumarin synthesis) and *PDR9* (related to coumarin transport) expression is under FIT control [45]. The regulation of *BGLU42* and *PDR9* expression by the EIN3/EIL1/FIT complex occurs through the MYB72 TF [46].

In relation to P deficiency responses, ET, through EIN3/EIL1, has also been involved in the regulation of several genes related to P acquisition from the soil, such as *PAP17, PHT1;1* and *PHT1;4*, and the P internal transporter *PHT1;5*. For activation of P acquisition genes, EIN3/EIL1 act through the PHR1 TF [69,99] while, to activate *PHT1;5* expression, EIN3/EIL1 act through the WRKY75 TF [135].

In a recent work, Feng et al. [136] showed that EIN3 physically interacts with ROOT HAIR DEFECTIVE 6 (RHD6), a positive regulator of hair cells, and that this complex directly coactivates the hair length-determining gene RHD6-LIKE 4 (*RSL4*) to promote root hair elongation. The results obtained from transcriptome analysis revealed the parallel roles of the regulator pairs EIN3/EIL1 and RHD6/RSL1 (RHD6-LIKE 1) and concluded that EIN3/EIL1 and RHD6/RSL1 coordinately enhance root hair initiation by selectively regulating a subset of core root hair genes.

In relation to the role of NO and GSNO in the regulation of different Fe acquisition processes, right now it is very difficult to discriminate between both compounds. However, in a recent work, by using a chemical screening approach, it has been proposed that NO and GSNO have different roles in the regulation of Fe acquisition genes [111].

**Figure 2:**
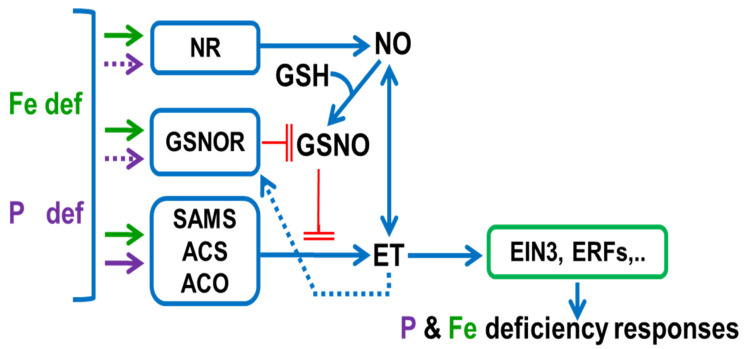
Model to summarize the relationship between ethylene, NO and GSNO in Fe- and P-deficient roots. Fe and P deficiency conditions induce several enzymes related to NO and ET synthesis, like NR, SAMS, ACS and ACO. Each one (NO, ET) mutually influences the synthesis of each other. Fe and P deficiency can also induce the GSNOR enzyme, which leads to a decrease in GSNO levels. GSNO, formed from NO and GSH, can limit ET synthesis through SAMS nitrosylation. On the other hand, ET can promote GSNO degradation by inducing NR and the GSNOR enzyme. Finally, ET, through EIN3 and other ERF TFs, would activate Fe and P deficiency responses. ET, ethylene; GSH, glutathione; GSNO, *S*-nitrosoglutathione; NO, nitric oxide. NR, nitrate reductase; GSNOR, GSNO reductase; SAMS, S-Adenosylmethionine synthetase; ACS, ACC syntase; ACO, ACC oxidase. →: promotion (dashed line indicates promotion based on preliminary results); ─╢: inhibition. Based on [112,138,142,143,144].

The positive and reciprocal influence between NO and ET in the regulation of Fe and Mg deficiency responses is already known [108,109,138]. However, the interrelationship between GSNO and ET is also feasible. It seems that the main way for GSNO to regulate Fe deficiency responses is through the reversible *S*-nitrosylation of proteins [139,140,141]. Among them are some enzymes involved in ET synthesis, such as SAM synthetases [142], which can be inhibited by *S*-nitrosylation [143,144]. In this way, higher GSNO levels (such as those found in Fe-sufficient roots) could contribute to *S*-nitrosylation of ET synthesis enzymes and, consequently, to the inhibition of ET synthesis [112], while lower GSNO levels (such as those found in Fe-deficient roots) could contribute to de-nitrosylation of ET synthesis enzymes and, consequently, to ET synthesis increase [112] (Figure 2).

Besides ET synthesis, GSNO (NO) could also affect ET signaling. Very recently, it has been found that the ERF72 TF (also named RAP2.3) participates in the regulation of Fe deficiency responses in *Arabidopsis* [132]. ERF72 belongs to the group VII ERFs [145], which are sensors of NO and can be targeted for proteolysis degradation by the N-end rule in the presence of NO [146]. Curiously, ERF72 interacts with DELLA proteins [145], also involved in the regulation of Fe deficiency responses [147].

GSNO/NO levels can be regulated by ET. In a recent work, García et al. [112] showed that ET (ACC) induces *GSNOR1* expression (Figure 2). ET (ACC) can also increase NO content by activating enzymes involved in its synthesis, such as nitrate reductase and nitric oxide synthase-like [138]. This would imply that ET could simultaneously increase NO accumulation and decrease GSNO content [112].

Although NO has also been studied in relation to P deficiency morphological responses [97,127], the role of endogenous GSNO and GSNOR in the regulation of P deficiency responses has hardly been studied [101].

## 7. Interactions with Other Signals in the Regulation of Fe and P Deficiency Responses

Besides activating signals, like ET and NO, in the regulation of Fe deficiency, responses have been involved inhibitory signals related to the Fe status inside the plant [112,148]. The existence of these repressive signals has been revealed by several experimental results showing that ET and NO activate the responses in plants growing with low levels of Fe but not in plants growing with high levels of Fe [105,106,108,114]. Later on, it has been shown that the signal(s) acting as inhibitor(s) is (are) related to the Fe that recirculates from shoots to roots through the phloem [112,148,149,150]. This statement is based on results obtained with the *Arabidopsis thaliana opt3-2* mutant, which is altered in a transporter involved in the loading of Fe into the phloem [150]. This mutant presents constitutive Fe deficiency responses, even under Fe sufficiency conditions [112,148,151]. Foliar Fe application does not have an inhibitory effect on their responses, contrary to what happens in the wild type [112,148], suggesting that the inhibitory signal could be a Fe-related compound associated with the OPT3 transporter [112,148,151].

Very recently, Balparda et al. [131] found that mutants in the PAP/SAL1 retrograde signaling pathway, altered in the communication between chloroplasts (and mitochondria) and the nucleus, are affected in the regulation of Fe deficiency responses in roots. Moreover, they have shown that there is a link between the PAP/SAL1 signaling pathway in leaves and the ET signaling and Fe deficiency responses in roots. These results agree with results described in the previous Section, showing a relationship between a Fe signal moving through the phloem and ET in roots. The 3′-phosphoadenosine 5′-phosphate (PAP) is a phosphonucleotide which accumulates, mainly in chloroplasts, in response to some abiotic stresses [152]. Under normal growth conditions, PAP is maintained at very low levels by the enzyme SAL1 phosphatase, which dephosphorylates it to adenosine monophosphate (AMP) and inorganic phosphate (Pi) [152,153]. SAL1 is in chloroplasts and mitochondria but not in the cytosol, although PAP can move between different cell compartments [152]. There are also results suggesting the presence of SAL1 in roots [154,155,156].

In relation to Fe, it has been found that *sal1* mutants present constitutive activation of Fe acquisition genes (*FIT, FRO2, IRT1*) in roots when grown under Fe-sufficient conditions [131]. Moreover, these authors showed that the constitutive activation of the Fe acquisition genes *FRO2* and *IRT1* was inhibited by ET inhibitors and, also, that *ERF1* expression was upregulated in Fe-sufficient roots of the *sal1* mutant. They also found, by using bioinformatic methods, that there are at least 9 ERF binding sites in the promoter region of both *FRO2* and *IRT1* genes [131]. ERF1 is a downstream component of the ET signaling pathway that is directly regulated by EIN3 (it also responds to JA) and that could integrate biotic and abiotic stress signals [129].

The relationship of PAP with ET could be related to its role as inhibitor of exoribonucleases (XRNs). XRNs (one of them is XRN4, also known as ET Insensitive 5: EIN5) participate in the ET signaling pathway and act as negative regulators of stress-inducible genes, like *ERF1* [131,152,157]. This is further supported by the fact that *xrn* mutants present upregulation of *ERF1*, and also of Fe acquisition genes, similarly to the *sal1* mutant [131]. The PAP/SAL1 pathway has also been related to other nutrients besides Fe, such as sulfur and P. The transcripts of several genes involved in sulfate assimilation and Pi uptake are upregulated in *sal1* mutants in the same way as in plants under sulfate or P starvation [155,158].

As with Fe acquisition genes, ET alone is not enough to induce the expression of P acquisition genes [57,121]. This suggests that, besides ET and NO, other P-related specific signals would participate in the regulation of P deficiency responses [57,121,159]. Again, a parallelism is found in the regulation of the responses to both deficiencies (Fe and P), suggesting the existence of specific repressor signals that would interact with ET/NO to activate or inhibit them. This hypothesis is supported by some experimental results obtained with the *Arabidopsis thaliana pho2* mutant, miss-regulated in P homeostasis [160]. The *pho2* mutant accumulates up to three more P in the aerial part than the wild type [160] and shows constitutive expression of P deficiency responses even under P sufficiency conditions, suggesting the lack of a specific repressor signal [161]. In the P case, the signal that could interact with ET/NO in the regulation of P deficiency responses could be the miRNA399, a P-related signal moving through the phloem [162,163]. It is overexpressed under P deficiency [164,165,166,167] and interacts with PHO2 to inhibit it [162]. *PHO2* encodes an ubiquitin-conjugating E2 enzyme and negatively regulates Pi uptake and root-to-shoot translocation [59,168]. The *pho2* mutant presents higher ACC (ET precursor) content in roots than the wild type when grown under P-sufficient conditions, which suggests that PHO2 could inhibit ET synthesis [59].

NO has also been involved in the regulation of lateral root development and inhibition of the primary root extension, typical responses to P deficiency. In this sense, a crosstalk between NO and auxin has been shown. NO accumulation was observed after indolacetic acid (IAA) exposure in cucumber plants, and treatments with NO donors (SNP and SNAP) induced adventitious root formation [169]. Similar results were observed in tomato and maize plants treated with IAA and NO donors [170,171]. SNP treatment strongly reduced primary root length in tomato and *Arabidopsis* plants [172,173]. Primary root growth inhibition under P deficiency can also be regulated through the interaction NO-gibberellins (GA). P deficiency inhibits GA biosynthesis in *Arabidopsis*, which acts as a positive regulator of the primary root growth [173,174]. NO seems to act by stabilizing DELLA proteins (negative regulators of GA signaling) in the nucleus [173]. The NO involvement in the regulation of the root hair development seems to be as a mediator of auxin action [174]. The involvement of auxin in the morphological changes under P deficiency has been widely studied. In a very recent work, a role for auxin in the regulation of P deficiency responsive gene expression has also been shown [175]. In that study, authors reported that *PHR1* is positively regulated by auxin signaling in *Arabidopsis* by means of the transcription factors AUXIN RESPONSE FACTOR7 (ARF7) and ARF19.

How plants perceive different deficiencies and translate this perception into the activation of physiological and morphological responses is unknown. However, the content of some P- or Fe-related molecules have been associated with the adequate levels of these nutrients. Inositol phosphates (IPs) have been associated with those of P deficiency responses [15,176] and Fe-peptides with those of Fe deficiency [112,177]. It has been shown that IPs can bind SPX proteins and stabilize the PHR1-SPX association to avoid the binding of PHR1 to its target [176]. Likewise, Fe peptides can bind BTS proteins and negatively regulate Fe deficiency responses [16,112,178]. From the Fe or P deficiency perception, until the altered expression of the target genes, several steps should occur in which hormones and signaling substances, like auxin, ET, cytokinins (CKs), NO, GSNO, ROS, sugars and miRNAs, have been involved [58,82,99,100,101,168,179,180]. Some of them, like auxin, ET and NO, have been described as activators of Fe or P deficiency responses [33,57,58,59,69,81,99,100,101,105,109,159,168,179,180,181], while other ones have been described as repressors, like CKs [182,183].

A clear understanding of Fe and P interactions is indispensable in order to improve the plant nutrition of both elements. Parallelisms and crosstalk, that could underpin the Fe/P interaction observed at the nutrient acquisition level, have been reported [58,81] but are not fully understood. ET seems to play a key role in the regulation of Fe and P deficiency responses since the EIN3/EIL1 TFs, acting in the final steps of the ET signaling pathway, can interact with several key TFs involved in the regulation of Fe and P deficiency responses, such as FIT, ERF1 and RHD6, related to Fe deficiency, and PHR1, WRKY75 and RHD6, related to P deficiency (Figure 1). NO/GSNO can also participate in the regulation of both Fe and P deficiency responses through their interactions with ET and other hormones, such as auxin.
